# Hybridization of long short-term memory neural network in fractional time series modeling of inflation

**DOI:** 10.3389/fdata.2023.1282541

**Published:** 2024-01-04

**Authors:** Erman Arif, Elin Herlinawati, Dodi Devianto, Mutia Yollanda, Dony Permana

**Affiliations:** ^1^Information system study program, Universitas Terbuka, Tangerang Selatan, Indonesia; ^2^Mathematics study program, Universitas Terbuka, Tangerang Selatan, Indonesia; ^3^Department of Mathematics and Data Science, Universitas Andalas, Padang, Indonesia; ^4^Department of Statistics, Universitas Negeri Padang, Padang, Indonesia

**Keywords:** inflation rate, ARFIMA, heteroscedasticity, ARFIMA-GARCH, ARFIMA-LSTM

## Abstract

Inflation is capable of significantly impacting monetary policy, thereby emphasizing the need for accurate forecasts to guide decisions aimed at stabilizing inflation rates. Given the significant relationship between inflation and monetary, it becomes feasible to detect long-memory patterns within the data. To capture these long-memory patterns, Autoregressive Fractionally Moving Average (ARFIMA) was developed as a valuable tool in data mining. Due to the challenges posed in residual assumptions, time series model has to be developed to address heteroscedasticity. Consequently, the implementation of a suitable model was imperative to rectify this effect within the residual ARFIMA. In this context, a novel hybrid model was proposed, with Generalized Autoregressive Conditional Heteroscedasticity (GARCH) being replaced by Long Short-Term Memory (LSTM) neural network. The network was used as iterative model to address this issue and achieve optimal parameters. Through a sensitivity analysis using mean absolute percentage error (MAPE), mean squared error (MSE), and mean absolute error (MAE), the performance of ARFIMA, ARFIMA-GARCH, and ARFIMA-LSTM models was assessed. The results showed that ARFIMA-LSTM excelled in simulating the inflation rate. This provided further evidence that inflation data showed characteristics of long memory, and the accuracy of the model was improved by integrating LSTM neural network.

## 1 Introduction

Big data are datasets of huge size or complexity, beyond the capabilities of conventional data-processing applications. While data with numerous entries holds higher statistical power, those with more attributes or columns can exhibit a larger false discovery rate. The term “big data” is now commonly used to describe the application of advanced data analytics models to massive data, rather than referring to a specific quantity. These models consist of predictive analytics, user behavior analysis, and other models that extract value from big data. In numerous fields including Internet searches, fintech, healthcare analytics, geographic information systems, urban informatics, and business informatics, massive datasets consistently pose challenges for scientists, corporate executives, medical practitioners, advertisers, and government officials.

Inflation refers to a data mining phenomenon that poses economic challenges for both governments and citizens in developing nations, including Indonesia. The issue of inflation represents a crucial indicator in preserving the stability of an economy (DeLong, 1997). According to Bank Indonesia, inflation can be defined as a general and sustained upsurge in the prices of goods and services over a specific period. It is important to note that an increase in the price of one or two goods alone does not constitute inflation, except this increase extends to other goods. When inflation escalates persistently and without control, it transforms into hyperinflation. The repercussions of hyperinflation reverberate negatively in the economic growth of a nation and bear an impact on the socioeconomic conditions of the populace. Additionally, this phenomenon complicates decision-making for economic stakeholders in taking the next step. The situation becomes further intricate when the domestic inflation rate surpasses those of other countries, which subsequently exerts pressure on the depreciating rupiah value. However, excessively low inflation also raises concerns, as prolonged low inflation can signal an economy operating below its potential capacity, leading to decreased economic growth and limiting the scope of monetary policies designed to bolster the economy. Inflation can be stated to be short-term and long-term, hence, it necessitates both anticipation and action to avert extreme highs and lows. The key to maintaining inflation within manageable bounds lies in the formulation of economic policies aimed at inflation control (Rahman et al., 2020).

By considering various factors in monetary policy, Bank Indonesia typically adjusts the benchmark interest rate in response to projected deviations from the established inflation target. To facilitate this forecast, predictions of inflation patterns play a vital role in preempting unstable macroeconomic conditions (Hasenzagl et al., 2022). These predictions often draw on mathematical sciences, employing time series models to analyze the movement of inflation data. Time series models arrange data chronologically and leverage the presumed repetition of patterns from past periods into the present and future. The purpose of time series model analysis is to uncover patterns for modeling future events (Alyousifi et al., 2021), identifying a variety of patterns that show to be influential on the response variable (Wu et al., 2023), enhancing forecasting accuracy and stability from the perspectives of noise distribution and outliers (Yang et al., 2023), and exploring how a particular model selection can be better applied to forecast the new data in the future (Xu et al., 2023). Based on the abundant data and its diverse attributes consisting of trends, seasonality, and cyclicality, current values are often modeled based on past data exhibiting inter-variable correlations, commonly through linear or nonlinear models.

Time series data patterns are typically categorized into short-term and long-term patterns. Short-memory patterned time series data feature weak correlations within short periods. The Auto-Regressive Integrated Moving Average (ARIMA) model serves to capture these patterns, requiring data to be stationary. Stationarity is achieved through differencing, where the differencing value (*d*) is a non-negative integer. The ARMA model, which is a fusion of the AR and MA models, combines autoregressive variables and past residues to model variable movement. ARIMA model excels in precise short-term forecasting and often indicates extended flat modeling periods.

In some instances, time series data exhibit robust correlations over a prolonged observation period. This is evident when autocorrelation values in the Autocorrelation Function (ACF) plot decline gradually over an extended span. In this aspect, the differencing value (*d*) becomes a real number, which is addressed by the Auto-regressive Fractionally Integrated Moving Average (ARFIMA) model (Huang et al., 2022). In 1980, Granger and Joyeux introduced ARFIMA model, an advancement of ARIMA model capable of predicting both short and long-memory-patterned time series data. This flexibility extends to the ability of ARFIMA to predict data exhibiting short-term as well as long-term memory patterns (Devianto et al., 2022). Subsequently, in 1981, Hosking examined the properties of long memory patterns in both stationary and non-stationary ARFIMA models. The Geweke and Porter-Hudak (GPH) model, which directly determines the fractional order value of differencing (*d*) without prior specification of significant AR and MA orders, offers a specific model for defining the fractional order value of differencing (*d*).

In the context of time series modeling, the application of the model hinges on the fulfillment of certain assumptions for the residuals. These assumptions entail non-autocorrelation, non-heteroscedasticity, and normality. Heteroscedasticity, a phenomenon in the model, typically arises due to the random fluctuations in the data, leading to fluctuating variance within the model. Addressing this issue necessitates an advanced strategy, as the variance in the model continues to fluctuate randomly. To address this challenge, the classical time series model known as the Generalized Autoregressive Conditional Heteroscedasticity (GARCH) is employed. However, it remains crucial to uphold the residual assumption. This model assists in estimating optimal parameters for nonlinear models through alternative iterative processes, resulting in accurate approximations of real values. A nonlinear model involves employing numerical or iterative processing on extensive data, enabling precise approximation of actual values. Among the popular nonlinear models, the neural network stands out. The neural network employs iterative processing, starting with random weight adjustments, followed by subsequent modifications to enhance initial weights (Yollanda et al., 2018). During the training of substantial data, initial weights are refined through the backpropagation model to correct errors. This model is combined with classical time series using the integer order model (ARIMA) and applied to counterbalance the residual of ARIMA model, which includes the heteroscedasticity effect (Devianto et al., 2023). In the domain of finance, the fractional order of ARIMA, known as ARFIMA, anticipates abrupt stochastic fluctuations within financial markets. In addition, it integrates the dynamic aspects of deep learning through Long-Short-Term Memory (LSTM) network (Bukhari et al., 2020).

LSTM model, belonging to the category of recurrent neural network, effectively overcomes the challenge of vanishing gradients that often arise in training. Its versatility allows for application across various topics, including the intricate and volatile financial market (Gajamannage et al., 2023), and specific cases such as asset pricing in the Chinese stock market (Pan et al., 2023), due to its ability to mitigate error propagation during iterations. Neural network also finds usefulness in predicting energy demand and *CO*_2_ emissions (Javanmard et al., 2023). In this study, a fusion of a multiobjective mathematical model with data-driven machine learning algorithms enhances the accuracy of energy demand and *CO*_2_ emissions forecasts in the transportation sector.

The implications for employing a statistical procedure, neural, and combined techniques for time-series forecasting of findings in the healthcare domain to forecast the expenditures of two different pain medications are demonstrated by combining ARIMA, neural network, and LSTM with the integer value of differencing (Kaushik et al., 2020). According to the studies related study, the suggested model requires to be verified by evaluating the residual assumption using a classical time series autoregressive moving average with either an integer or fractional differencing order. Since the inflation data fluctuates randomly over time, the model typically exhibits heteroscedasticity. This study makes use of a long short-term memory neural network (LSTM) and generalized autoregressive conditional heteroscedasticity (GARCH) to provide some alternative suggested techniques to improve the sensitivity of heteroscedasticity effects present in residual classical time series data with long-memory patterns.

## 2 Materials and methods

### 2.1 Data source

The study included datasets of 162 monthly inflation records, spanning from February 2009 to July 2022. The data source was available on the Bank Indonesia website, specifically at https://www.bi.go.id.

### 2.2 Long-memory time series of autoregressive fractional integrated moving average

Managing big data required employing numerical processing to identify data patterns and approximate optimal parameters. Various statistical models were used to address fluctuations rooted in data patterns. An example of this model was time series analysis, used to scrutinize data mined over time, often involving significant data volumes. This model aimed to forecast future events and comprehend underlying processes by studying data patterns and trends. Time series analysis found applications in diverse fields, including forecasting consumer demand, predicting stock market shifts, and understanding economic indicators such as inflation and unemployment trends. These models assisted in comprehending trends, seasonality, and noise within data, using past data to predict future values. Furthermore, time series analysis could predict the impact of exogenous factors, namely governmental policy changes or technological advancements. Various elements could have an impact on a time series (Gulmez, 2023):

- Trend: Refers to an entire direction, whether ascending, descending, or stable, that a time series displays over time.
- Seasonality: Denotes recurring trends within data, such as heightened sales during holidays.
- Cyclicality: Implies the presence of long-term tendencies such as economic booms and busts.
- Irregularity: Accounts for random or unforeseen fluctuations in data.
- Exogenous factors: Entail external influences, namely monetary policies, crises, or societal shifts.

To create a time series model incorporating long memory elements, ARFIMA model follows a series of stages:

**Step 1**. Checking the stationarity of the data with respect to variance. Let {*X*_*t*_} be a time series data sequence. Since the data are not stationary, data transformation was performed to obtain a rounded value (λ) using Box-Cox transformation *T*. For example, assuming data *X*_*t*_ is non-stationary regarding variance, it can be transformed with the formula $T\left(X_{t}\right)=\left(X_{t}^{\lambda -1}\right)/\lambda$, where λ is the transformation parameter. Stationarity is achieved when λ = 1 yields a rounded value process (Devianto et al., 2022).

**Step 2**. Checking the stationarity of the data with respect to mean by using Augmented Dickey-Fuller (ADF) test. The random walk equation with drift for the differenced-lag model is regressed to be:


(1) $$\begin{array}{l} \nabla X_{t}=\mu +\delta X_{t-1}+\overset{k}{\underset{i=1}{\sum }}\phi_{i}\nabla X_{t-i}+e_{t} \end{array}$$

for ∇*X*_*t*_ = *X*_*t*_ − *X*_*t*−1_, *k* is the number of lags, δ is the slope coefficient, μ is a drift parameter, ϕ_*i*_ is parameter of random walk equation, and *e*_*t*_ is white noise error term. The test statistic is used as follows:


(2) $$\begin{array}{l} ADF=\frac{\hat{\delta }}{SE\left(\hat{\delta }\right)} \end{array}$$

for $\hat{\delta }$ as the estimated δ which is obtained by using ordinary least squares and $SE\left(\hat{\delta }\right)$ as the standard error of δ. The initial hypothesis is δ = 0, which means that the data is not stationary. The criteria for decision-making reject the initial hypothesis if the ADF value is less than the test statistics in the table.

**Step 3**. As graphically, plotting the autocorrelation function (ACF) of the transformed data in Step 1 to detect the presence of a long-memory effect. If the ACF pattern is generated as a sine function, there is a long-memory pattern data. As mathematically, long-memory pattern data can be used the differencing parameter *d* explained in Step 4.

**Step 4**. Estimating the differentiating parameters *d* using the Geweke and Porter-Hudak model, denoted as ${\hat{d}}_{GPH}$ with the following formula (Devianto et al., 2022):


(3) $$\begin{array}{l} {\hat{d}}_{GPH}=\frac{\sum_{j=1}^{m}\left(x_{j}-\bar{x}\right)\left(y_{j}-ȳ\right)}{\sum_{j=1}^{m}{\left(x_{j}-\bar{x}\right)}^{2}} \end{array}$$

where *y*_*j*_ = *lnI*(λ_*j*_) and $x_{j}=-ln{\left[2sin\left(\frac{\lambda_{j}}{2}\right)\right]}^{2}$. The *I*(λ_*j*_) function is a periodogram with a frequency of Fourier $\lambda_{j}=\frac{2\pi j}{T}$, *j* = 1, 2, ⋯   , *m*, and *T* is the number of observation data while *m* is the limit of the number of Fourier frequencies.

**Step 5**. Transforming the differential data using the obtained ${\hat{d}}_{GPH}$ values.

**Step 6**. Identifying potential ARFIMA model by combining significant orders of autoregressive (AR) and moving average (MA) models using PACF and ACF plots of stationary data, respectively.

**Step 7**. Estimating parameters and testing the significance of ARFIMA model. Parameter estimation is performed on each model, followed by significance tests. A model is considered feasible when its parameters are significant, with probability values smaller than α = 5%.

**Step 8**. Selecting the best ARFIMA model based on the smallest Information Criterion (AIC) value of Akaike.

**Step 9**. Testing the residual assumptions of the best ARFIMA model, including non-autocorrelation and normality.

**Step 10**. Determining the best ARFIMA model equation and its interpretation.

A time series *X*_*t*_ is classified as a sequence of white noise when an uncorrelated random variable, adhering to a specific distribution, maintains a constant mean of zero and a constant variance of $Var\left(X_{t}\right)=sigma^{2}$ along with *Cov*(*X*_*t*+*h*_, *X*_*t*_) = 0 for *k* ≠ 0. Among classical time series, ARIMA combines AR and MA models after integer differencing. ARIMA evolved into ARFIMA, which integrates ARFIMA features. ARFIMA parallels the structure of ARIMA but relies on fractional values for differencing, as opposed to ARIMA integer differencing. Let ϕ_*p*_(*B*) as AR components, θ_*q*_(*B*) as MA components, *B* as the operator of backward shift, and ${\left(1-B\right)}^{d}X_{t}$ indicates the *d*-order differenced stationary time series, the process is labeled ARFIMA(p, d, q) (Devianto et al., 2023):


(4) $$\begin{array}{l} \phi_{p}\left(B\right){\left(1-B\right)}^{d}X_{t}=\theta_{q}\left(B\right)\varepsilon_{t} \end{array}$$

with


$$\begin{array}{l} \phi_{p}\left(B\right)=\left(1-\phi_{1}B-\phi_{2}B^{2}-\cdots -\phi_{p}B^{p}\right)\\ \theta_{q}\left(B\right)=\left(1-\theta_{1}B-\theta_{2}B^{2}-\cdots -\theta_{q}B^{q}\right) \end{array}$$

where *p*, *q*, and *B* are the positive integer values.

ARFIMA model that had been fitted was then extended into time series analysis to determine whether the residual assumptions were met. It should be noted that these assumptions began with autocorrelation and further consisted of heteroscedasticity and normality. The initial hypothesis assumed that there was no linear relationship in dependency within the residual ARFIMA model. The statistic value *Q*_*LB*_ could be expressed as follows (Devianto et al., 2023):


(5) $$\begin{array}{l} Q_{LB}=n\left(n+2\right)\overset{k}{\underset{i=1}{\sum }}\frac{\rho_{i}^{2}}{n-i} \end{array}$$

where *n* is the number of data, the sample auto-correlation coefficient at lag *k* = 1, 2, 3, ⋯   , *K* is denoted as $\rho_{i}^{2}$, and lag length is denoted as *K*. The initial hypothesis was rejected when the statistic value was greater than the critical value or $Q_{LB}>\chi_{\alpha }^{2}\left(k-p-q\right)$. On the other hand, the hypothesis could be rejected assuming the probability value was less than the significance level.

The second assumption concerned the heteroscedasticity effect using the Lagrange Multiplier (LM) test by White. The initial hypothesis of the LM test assumed that the residual ARFIMA model exhibited homoscedasticity, where the variance of this residual model remained constant, allowing the random fluctuated data to be ignored. The statistic value of the *LM* test was the product of the determination coefficient value *R*^2^ and the sample size *n*, expressed as follows:


(6) $$\begin{array}{l} LM=nR^{2} \end{array}$$

The LM test followed a chi-squared distribution with degrees of freedom equal to *k* − 1, where *k* was the number of estimated parameters. The initial hypothesis was rejected because the statistic value was greater than the critical value $LM>\chi_{\alpha }^{2}\left(k-1\right)$. However, the hypothesis could be rejected when the probability value was less than the significance level.

The final assumption was the normality test, where the initial hypothesis stated that the residual skewness (*S*) and kurtosis (*K*) of ARFIMA model matched a normal distribution with expected values of zero. This was determined using the Jarque-Bera (JB) test, and the statistics test of *JB* could be expressed as follows (Devianto et al., 2023):


(7) $$\begin{array}{l} JB=\frac{n}{6}\left(S^{2}+\frac{{\left(K-3\right)}^{2}}{4}\right) \end{array}$$

where *K* and *S* are kurtosis and skewness, respectively. The initial hypothesis was rejected since the statistic value was greater than the critical value $JB>\chi_{\alpha }^{2}\left(2\right)$ or when the probability value was less than the significance level of 5%.

When fitting ARFIMA model, potentially significant models were selected, and the best model was selected. The goodness-of-fit criteria such as AIC, BIC, and HQ were applied, using the loglikelihood function to determine the best model. Let ${\hat{\sigma }}_{\varepsilon }^{2}$ represent the maximum likelihood estimator of $\sigma_{\varepsilon }^{2}$, *k* denoted the number of estimated parameters, and *n* indicated the number of observations. The equations for AIC, BIC, and HQ were systematically written as follows:


(8) $$\begin{array}{l} AIC=nln\left({\hat{\sigma_{\varepsilon }}}^{2}\right)+2k \end{array}$$

(9) $$\begin{array}{l} BIC=nln\left({\hat{\sigma_{\varepsilon }}}^{2}\right)+kln\left(n\right) \end{array}$$

(10) $$\begin{array}{l} HQ=nln\left({\hat{\sigma_{\varepsilon }}}^{2}\right)+2kln\left(ln\left(n\right)\right) \end{array}$$

The best model was selected based on the smallest value among AIC, BIC, and HQ. Assuming there were two smallest values in any of these criteria, a nonparametric model was required to determine the smallest rank.

### 2.3 Improving volatility sensitivity with classical generalized autoregressive conditional heteroschedasticity

Time-series analysis involved four factors, namely trend, seasonality, periodicity, or cycle, as well as irregular components, all of which generally contributed to the variations in the data. In 1982, Engle introduced ARCH, assuming that the data variance was influenced by past values. Let *Q* denote the number of autoregressive terms in the model, t represents the ordinary least squares variance obtained from the original regression model Equation (4), α_*i*_ stands for a parameter of ARCH, ε_*t*_ = σ_*t*_*e*_*t*_, and *e*_*t*_ ~ *N*(0, 1) with α_*i*_ > 0. The variance assumption of ARCH and ARIMA in Equation (4) allowed ARCH(*Q*) to be stated as follows (Devianto et al., 2023):


(11) $$\begin{array}{l} \sigma_{t}^{2}=\alpha_{0}+\alpha_{1}\varepsilon_{t-1}^{2}+\cdots +\alpha_{Q}\varepsilon_{t-Q}^{2} \end{array}$$

for *i* = 0, 1, 2, ⋯   , *Q*. While processing real data, ARCH often produced higher orders, leading to an increase in the number of estimated parameters. In order to replace GARCH model as the preferred one, ARCH model was created. The residual *e*_*t*_ was assumed to be homoscedastic since it represented the concept of a time series, i.e., $E\left(e_{t}^{2}\right)=E\left(e_{t}^{2}|e_{t-1}^{2},e_{t-2}^{2},\cdots \;\right)=\sigma^{2}$ for each *t*. However, the variance of *e*_*t*_ fluctuated over time in GARCH model, resulting in $E\left(e_{t}^{2}|e_{t-1}^{2},e_{t-2}^{2},\cdots \;\right)=\sigma_{t}^{2}$, which indicated heteroscedasticity.

GARCH model required that the initialized data had constant variances. In simpler terms, the fluctuations in the data did not always have the same value at time *t*. An *e*_*t*_ process was considered a GARCH(*P*,*Q*) process when it satisfied the following conditions:


(12) $$\begin{array}{l} \sigma_{t}^{2}=\alpha_{0}+\overset{Q}{\underset{i=1}{\sum }}\alpha_{i}\varepsilon_{t-i}^{2}+\overset{P}{\underset{j=1}{\sum }}\beta_{j}\sigma_{t-j}^{2} \end{array}$$

where ε_*t*_ = σ_*t*_*e*_*t*_, *e*_*t*_ ~ *N*(0, 1), *Q* > 0, *P* ≥ 0, α_*i*_ ≥ 0, α_0_ > 0, for *i* = 0, 1, 2, ⋯   , *Q* and β_*j*_ ≥ 0 for *j* = 1, 2, ⋯   , *P* and also $\overset{Q}{\underset{i=1}{\sum }}\alpha_{i}+\overset{P}{\underset{j=1}{\sum }}\beta_{j}<1$.

The Maximum Likelihood Estimation (MLE) model was then used to initially approximate GARCH parameters. White noise with a mean value of 0 and a variance of σ^2^ was thought to make up the residual of ARIMA model. The parameters were estimated using iterative procedures, such as Newton-Raphson, after obtaining the log-likelihood function for *n* observed data points. This model was used to solve the probability log function. Consequently, an estimator that sufficiently converged for each parameter was obtained.

### 2.4 Improving volatility sensitivity with long short-term memory

**Figure 1:**
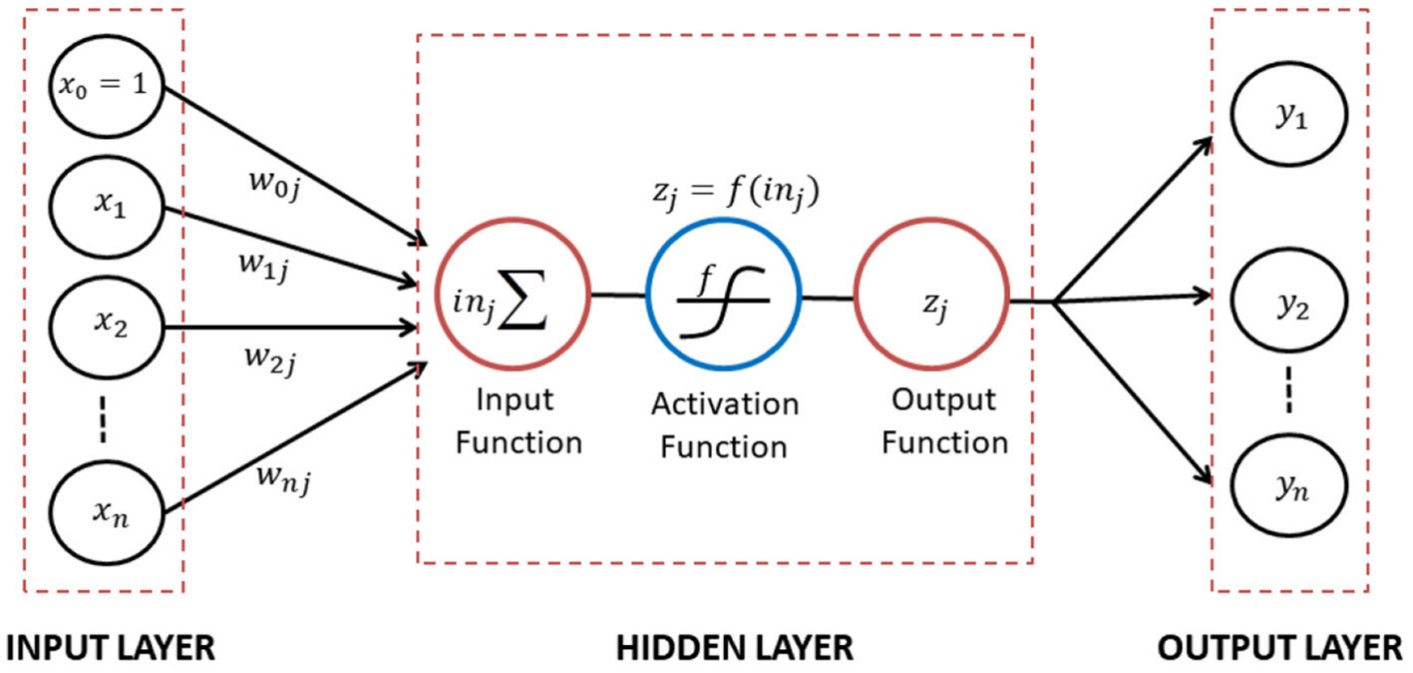
A diagram construction of mathematical neural network model.

In the context of big data analysis, it was possible to forecast the future by identifying patterns in past data. Additionally, a correlation existed between the variables and the historical data or data residuals. The artificial neural network was a system that mimicked the network of nerve cells in human and animal brains (Yollanda et al., 2018). Furthermore, it consisted of a few hidden layers that connected the input to the output layer. The connections between two layers were defined by weights and biases, also referred to as the coefficient of network. The construction of the mathematical neural network model was generally depicted in Figure 1 (Devianto et al., 2023).

Figure 1 showed the construction of neural network that connected two layers through directed links of weights or biases to determine the sign and strength of the input data. The input data *x*_*i*_, representing *x*_1_, *x*_2_, ⋯   , *x*_*n*_ was then calculated as a weighted sum of its inputs on the hidden layer, *in*_*j*_. Each hidden unit *j* transformed the input through the activation function *f*, yielding *z*_*j*_, expressed as follows:


(13) $$\begin{array}{l} z_{j}=f\left(in_{j}\right)=f\left(\overset{n}{\underset{i=0}{\sum }}x_{i}w_{ij}\right) \end{array}$$

This result was then passed as input to other neurons and the activation function *f* scaled the output *z*_*j*_ into appropriate ranges. The network architecture could be classified as feed-forward (FFNN) and recurrent neural network (RNN), commonly used for forecasting. In a feed-forward network, each input unit received data from the layer below. Meanwhile, the outputs of a recurrent network became inputs in the preceding layer. The recurrent network created a dynamical system where inputs depended on the initial values of previous inputs, in order to simulate a stable state (Devianto et al., 2023).

The feed-forward neural networks (FFNN) require one or more input unit(s) in processing in the hidden layer so that the results will have the output unit. The output unit is then compared to the observational or actual data as the supervised learning. In the time series case, there is no explanation which variable that will be the input or output units so that the time series model have separated one dataset to be two or more dataset by using the significant lag in the autocorrelation functions. Therefore, there is one type of recurrent neural networks that can be used to process the time series model. A common type of recurrent neural network was LSTM, designed for analyzing time series data. LSTM was equipped to handle long-term dependencies present in time series data, ensuring outcomes depended on previous data values. The LSTM model has the advantage of processing and predicting time series events with long intervals and delays. It means that scientists can use previous data to forecast new data in a few periods ahead. It limits applications of FFNN to time series models. FFNN has to receive input unit values before calculating the estimated output unit.

**Figure 2:**
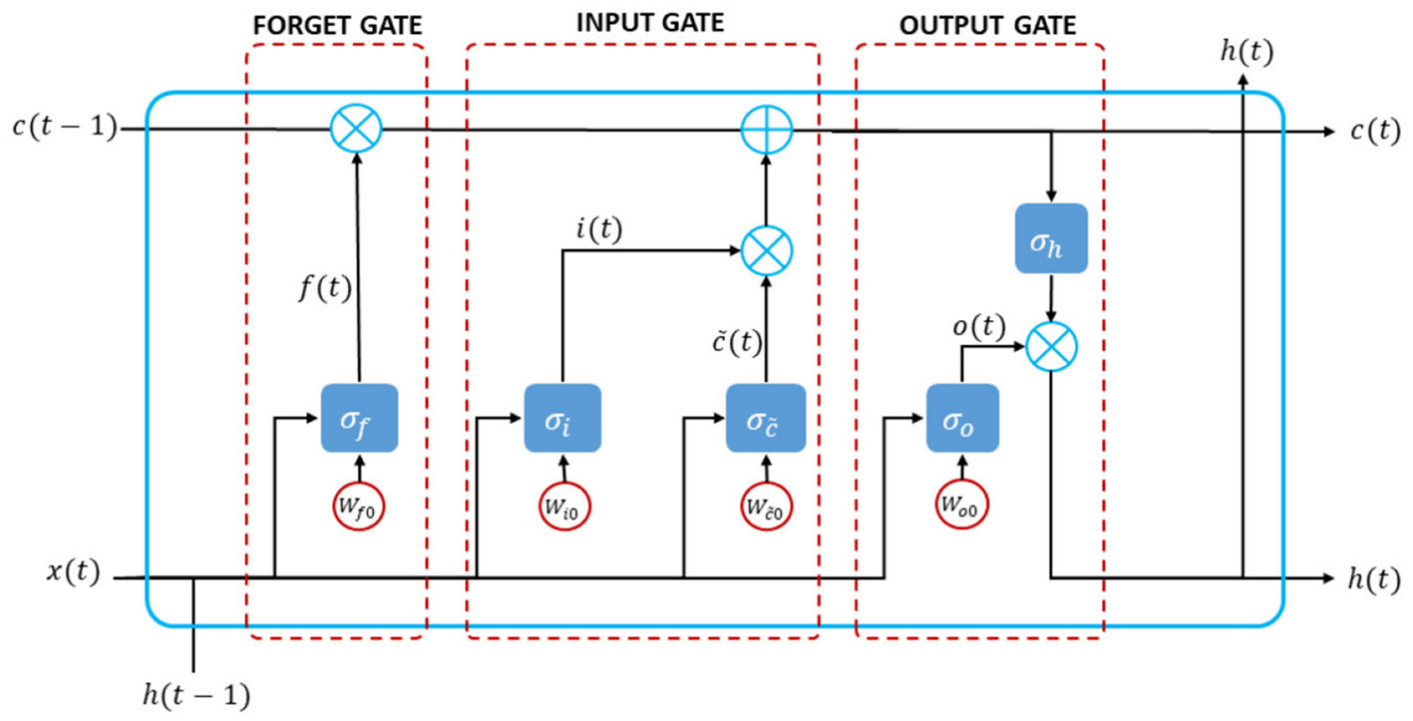
A diagram construction of mathematical long short-term memory neural network model.

In constructing the structural LSTM model, LSTM neural network comprised input gates, memory cells, forget gates, and output gates. These components processed information over longer periods. The network could selectively store and retrieve information as needed, regulated by these gates that controlled the flow of data into and out of memory cells. The input gate introduced new inputs to the cell, the forget gate maintained values for later use, and the output gate determined the output of the cell. Common tasks for which LSTM were employed included language translation, speech recognition, and stock price prediction. While RNN could learn long-term dependencies in data, it was impacted by the vanishing gradient problem. LSTM addressed this issue by using a set of gates to determine the data to retain and those to discard. This allowed the model to retain more information compared to RNN, achieved through gradient control (Haider et al., 2019). A typical LSTM cell structure could be seen in Figure 2.

The mathematical expressions for the inner connections of the gates in LSTM cell structure were as follows. Let *f*(*t*), *i*(*t*), *c*(*t*), and *o*(*t*) as forget gate, input gate, modulation cell gate, and output gate, respectively, with their activation functions of σ_*f*_, σ_*i*_, σ_*c*_, and σ_*o*_. In the modulation cell gate, the input is updated at the present time instant. All gates work on current input *x*(*t*) and previous values of states *h*(*t* − 1). As further, the following steps show how to design the Long Short-Term Memory (LSTM) neural networks:

1. Load time series dataset *X*(*t*) into the networks as the current input unit.
2. Normalize the dataset *x*(*t*). The scale of the input data impacts LSTMs, especially since the sigmoid (default) or tanh activation functions are employed. It is recommended to rescale the data to the 0-to-1 range as new minimum and new maximum dataset, respectively, also known as normalizing. The normalization of the dataset can be expressed as follows:
  (14) $$\begin{array}{l} x(t)=\frac{X(t)-\min(X(t)))}{\max(X(t)))-\min(X(t)))}(\text{new }\max(X(t)))\\ \;-\text{new }\min(X(t))))+\text{new }\min(X(t))) \end{array}$$
  (15) $$\begin{array}{l} \;=\frac{X(t)-\min(X(t)))}{\max(X(t)))-\min(X(t)))}(1-0)+0\\ \;=\frac{X(t)-\min(X(t)))}{\max(X(t)))-\min(X(t)))} \end{array}$$
3. Split the dataset to be training and testing data. The sequence of values is important since it deals with time series data. You are able to split the ordered dataset into train and test datasets using a simple method. The networks separated the dataset into 70% for training the model and 30% for validating the testing data against the training data.
4. Reshape the training and testing data as input units (training X and testing X) and as output unit (training Y and testing X) based on the significant lag in autocorrelation function or first lag as the default.
5. Design and fit the LSTM neural networks. In this step, the number of iterations, input unit, hidden layers and their units, and output units are determined. The default sigmoid activation function is used for the LSTM blocks or units.
6. Reproduce the optimal weights for each unit. Let *W*_*jh*_, *W*_*jx*_, and *W*_*j*0_ as the weights for previous hidden unit, input unit, and bias, respectively for $j=f,i,\tilde{c}$ and *o*. The forget and input gates are calculated as follows (Yu et al., 2019):
  (16) $$\begin{array}{l} f\left(t\right)=\sigma_{f}\left(W_{fh}h\left(t-1\right)+W_{fx}x\left(t\right)+W_{f0}\right) \end{array}$$
  (17) $$\begin{array}{l} i\left(t\right)=\sigma_{i}\left(W_{ih}h\left(t-1\right)+W_{ix}x\left(t\right)+W_{i0}\right) \end{array}$$
  The modulation cell gate value was calculated as:
  (18) $$\begin{array}{l} \tilde{c}\left(t\right)=\sigma_{\tilde{c}}\left(W_{\tilde{c}h}h\left(t-1\right)+W_{\tilde{c}x}x\left(t\right)+W_{\tilde{c}0}\right) \end{array}$$
  (19) $$\begin{array}{l} c\left(t\right)=f\left(t\right)c\left(t-1\right)+i\left(t\right)\tilde{c}\left(t\right) \end{array}$$
  The current cell state, current input, and previous state of the cell were used to control the output gate. The updated output and hidden cell state were given as follows:
  (20) $$\begin{array}{l} o\left(t\right)=\sigma_{o}\left(W_{oh}h\left(t-1\right)+W_{ox}x\left(t\right)+W_{o0}\right) \end{array}$$
  (21) $$\begin{array}{l} h\left(t\right)=o\left(t\right)\sigma_{h}\left(c\left(t\right)\right) \end{array}$$
  The design of deep network generally involved multiple hidden layers. To achieve the best results in the proposed work, several LSTM hidden layer topologies were evaluated. The optimal weights are obtained after optimizing the function.
7. Validate the LSTM neural networks model. The LSTM model is evaluated in this step to determine whether it is proposed or requires to be improved. If the error of the training data is greater than the error of the testing data, the processing is complete. However, iterations are continued if the training data has a smaller error than the testing data. It is necessary to determine whether the LSTM model can recognize unexpected or new data.
8. Calculate the evaluation model of Mean Squared Error (MSE), Mean Absolute Percentage Error (MAPE), and Mean Absolute Error (MAE).

### 2.5 Model evaluation

In modeling, evaluation criteria were used to assess how well it predicted output values from input data (Gulmez, 2023). The Mean Squared Error (MSE) measured the gap between actual values and projected values. It was calculated by averaging squared differences between expected and actual numbers. A lower MSE value indicated a more accurate model.


(22) $$\begin{array}{l} MSE=\frac{SSE}{n}=\frac{\sum_{t=1}^{n}{\left(y_{t}-\hat{y_{t}}\right)}^{2}}{n} \end{array}$$

The accuracy of a model was quantified by the Mean Absolute Percentage Error (MAPE), expressed as a percentage. It was computed by dividing the absolute difference between expected and actual values by the actual value, then averaging these percentages and calculating the difference between predicted and actual values. A lower MAPE number indicated better real-world prediction by the model.


(23) $$\begin{array}{l} MAPE=\frac{1}{n}\overset{N}{\underset{t=1}{\sum }}\frac{\left|y_{t}-\hat{y_{t}}\right|}{y_{t}}\times 100\% \end{array}$$

Another model to measure the gap between expected and actual values was the Mean Absolute Error (MAE). This was calculated by finding the absolute difference between expected and actual numbers, and then taking their average. The MAE statistic also assessed the model accuracy, with lower MAE values indicating higher results.


(24) $$\begin{array}{l} MAE=\frac{1}{n}\overset{N}{\underset{t=1}{\sum }}|y_{t}-\hat{y_{t}}| \end{array}$$

After calculating the optimal model, the ratio of sums of squares of regression to sums of total squares determined the coefficient *R*^2^. This measurement ranged from 0 to 1, and the value of *R*^2^ close to 0 indicated the estimated model did not fit well, while a value close to 1, implied it was well-fit. Let y be the mean of the dataset *y*_*i*_, where *i* = 1, 2, ⋯   , *n*. *R*^2^ was calculated as follows:


(25) $$\begin{array}{l} R^{2}=\frac{\sum_{i=1}^{n}{\left(\hat{y_{i}}-ȳ\right)}^{2}}{\sum_{i=1}^{n}{\left(y_{i}-ȳ\right)}^{2}} \end{array}$$

The value of *R*^2^ represented the amount of variance in the response variable explained by the predictor variable. Furthermore, it represented the squared correlation between observed values *y*_*i*_ and anticipated values $\hat{y_{i}}$ based on data processing.

## 3 Main results

This study used 162 data points of inflation in Indonesia on a monthly basis. This section involved building a long-memory pattern of ARFIMA model, enhancing volatility residual using LSTM model, and evaluating the preferred model using certain criteria.

### 3.1 Building long-memory pattern of ARFIMA model

**Figure 3:**
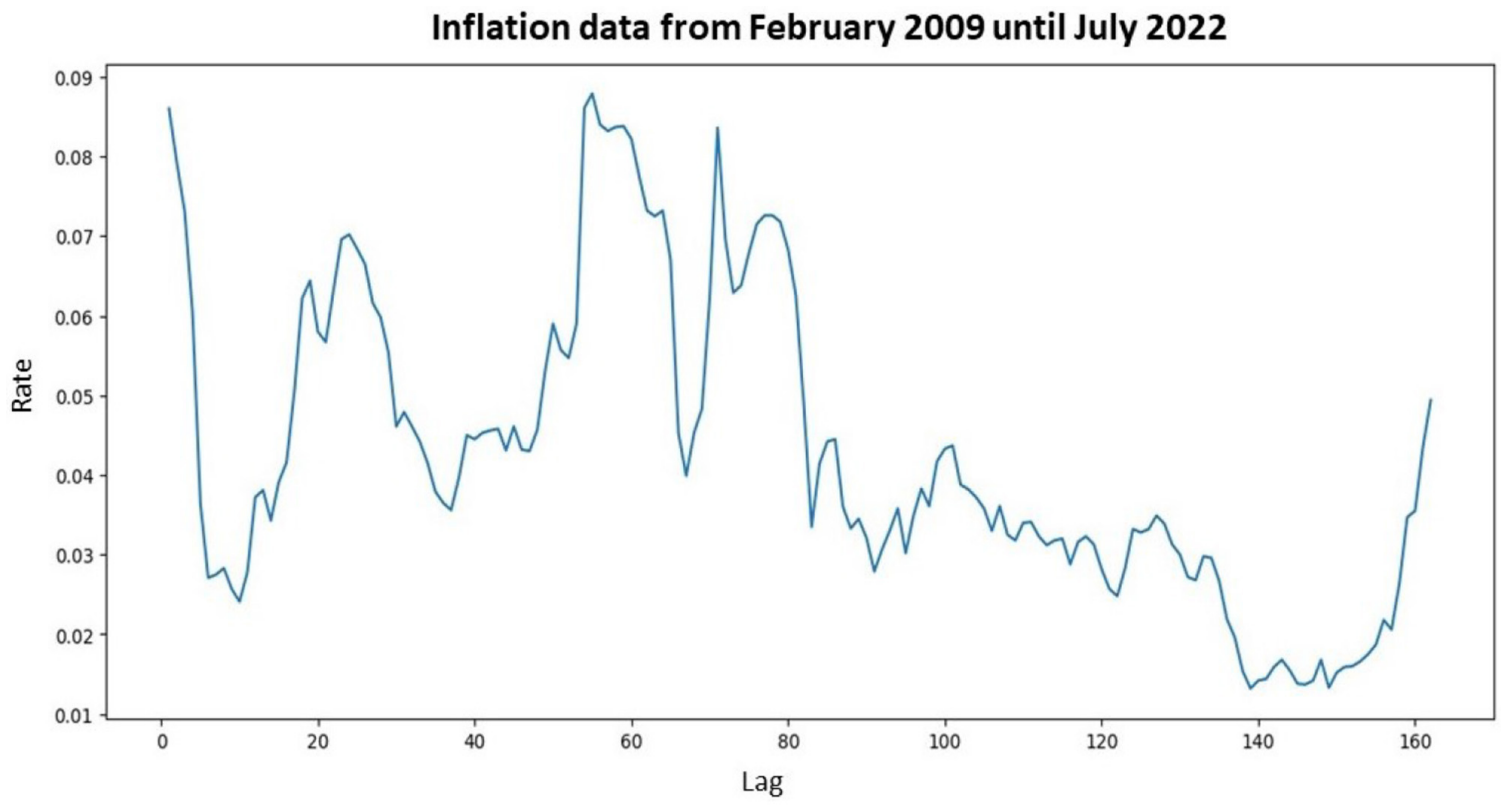
Plot of inflation data that starting from February 2009 until July 2022.

In this subsection, the modeling process was initiated with the initial step of identifying the movement of inflation data. The inflation data model with a monthly period was graphically shown in Figure 3.

Based on Figure 3, it could be concluded that the inflation data pattern for each period exhibited free fluctuations. Since the inflation data did not fluctuate around a constant mean and variance, the data were not stationary concerning mean and variance values. To address this non-stationarity in time series data regarding variance, data transformation was performed using the Box-Cox transformation. The first step was determining the rounded value (λ). Based on the transformation parameter formula, the value of λ was −14.57178, indicating non-stationarity with respect to variance. Therefore, a second-stage transformation was conducted with a λ value of 1, rendering the inflation data stationary regarding variance. The subsequent step was to check whether the data were stationary concerning mean values using the Augmented Dickey-Fuller test, shown in Table 1.

**Table 1:** Augmented Dickey-Fuller test.

**Critical value**	**ADF test**	
	**Statistics value**	***p*** **-value**
1%:−3.4722	−2.4232	0.1353
5%:−2.8799		
10%:−2.5766		

Lag order: 2.

**Figure 4:**
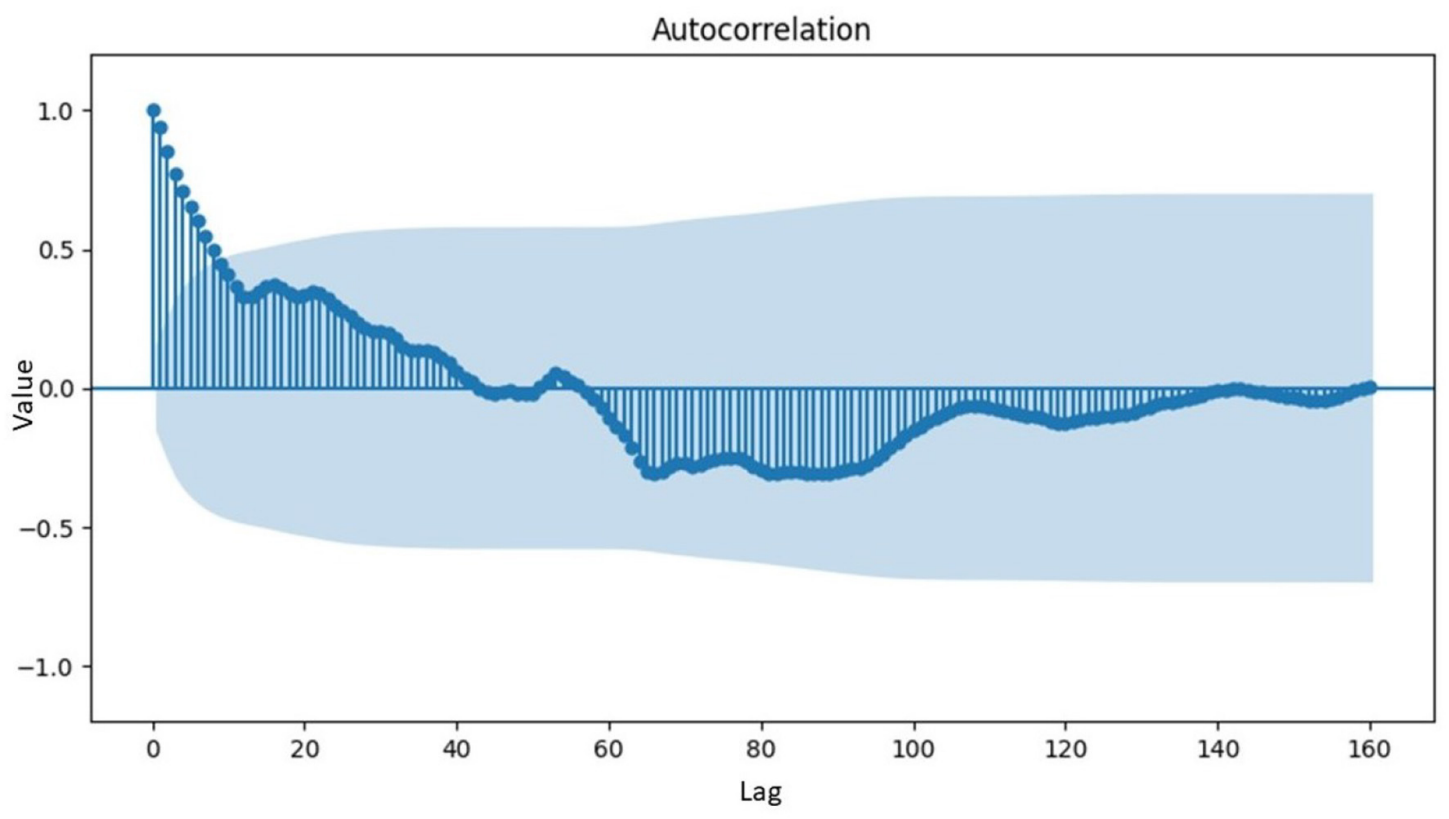
ACF plot of inflation data exhibiting variance stationarity.

From Table 1, the value of the statistic exceeded the critical value at the 5% significance level, implying that the data were not stationary concerning the mean value. This was further supported by the probability value of 0.1353, which was higher than the 5% significance level. To determine the order differencing of fractional or integer, the identification pattern used the Autocorrelation Function (ACF) to ascertain the presence of long-memory terms as shown in Figure 4.

**Figure 5:**
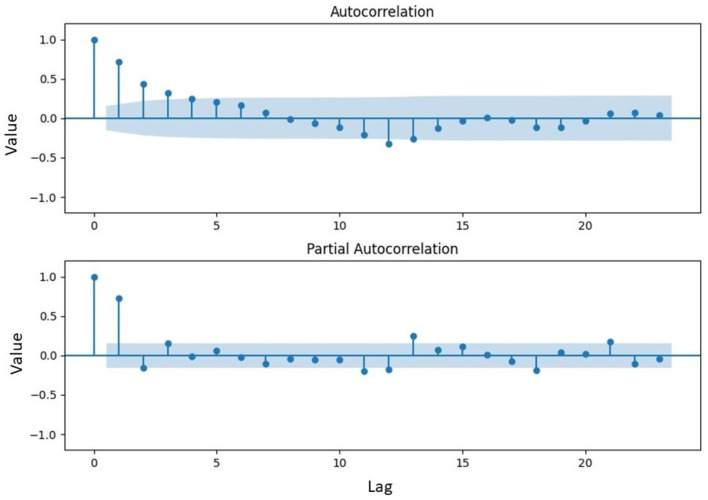
Plot of autocorrelation and partial autocorrelation function.

Figure 4 showed a gradual decrease in data over time, indicating the presence of a long-memory pattern and suggesting a fractional order differencing for the model. Mathematically, differencing with an order value of *d* was required to make the data stationary concerning the mean, estimated using the Geweke Porter-Hudak (GPH) model. The order model determined by the GPH model was ${\hat{d}}_{GPH}=0.4941$. As ${\hat{d}}_{GPH}=0.4941$ was <0.05, the data exhibited a long-memory effect and could be modeled with ARFIMA. Subsequently, the order of ARFIMA model was determined by identifying the number of significant lags in the ACF and PACF plots, as shown in Figure 5.

Based on Figure 5, the ACF coefficient reached a significant value at a lag of 5, while the PACF coefficient reached a significant value at a lag of 2. This suggested the possibility of forming an ARFIMA model by combining a maximum lag of 2 for parameter *p* and a maximum lag of 5 for parameter *q*, along with a *d*_*GPH*_ value of 0.4941. Furthermore, the parameters for each model were estimated and from the results, a significance test was conducted. The probability values for each model were shown in Table 2. A model was considered significant when the probability value of its parameter was <0.05.

**Table 2:** The significant estimated parameters of the ARFIMA model with their AIC or BIC value.

**Model**	**Estimation parameter**				**Model selection**	
	**Parameter**	**Estimate**	**Statistic**	**Pr(**>|*z*|**)**	**AIC**	**BIC**
ARFIMA(0, 0.4941, 1)	θ_1_ [MA(1)]	−0.7032	−15.0857	<2.22e-16	−1653.5420	−1641.1900
ARFIMA(0, 0.4941, 2)	θ_1_ [MA(1)]	−0.9077	−12.5615	<2.22e-16	−1675.3520	−1659.9100
	θ_2_ [MA(2)]	−0.3414	−5.4321	6.4070e-08		
ARFIMA(0, 0.4941, 3)	θ_1_ [MA(1)]	−0.9137	−11.5841	<2.22e-16	−1678.3420	−1659.8200
	θ_2_ [MA(2)]	−0.4605	−5.3404	1.0573e-07		
	θ_3_ [MA(3)]	−0.1701	−2.2116	0.0278		
ARFIMA(0, 0.4941, 4)	θ_1_ [MA(1)]	−0.9319	−11.7777	<2.22e-16	−1681.1090	−1659.5000
	θ_2_ [MA(2)]	−0.5003	−4.7312	2.4497e-06		
	θ_3_ [MA(3)]	−0.2863	−3.1530	0.0017		
	θ_4_ [MA(4)]	−0.1724	−2.2212	0.0270		
ARFIMA (1, 0.4941, 0)	Φ_1_ [AR(1)]	0.7571	−14.4049	<2.22e-16	−1681.1800	−1668.8300
ARFIMA(1, 0.4941, 1)	Φ_1_ [AR(1)]	0.6141	−7.2804	4.5617e-13	−1686.4910	−1671.0500
	θ_1_ [MA(1)]	−0.3212	−3.0764	0.0021		
ARFIMA(1, 0.4941, 3)	Φ_1_ [AR(1)]	−0.8938	−12.1332	<2.22e-16	−1674.3120	−1652.7000
	θ_1_ [MA(1)]	−1.9758	−17.0232	<2.22e-16		
	θ_2_ [MA(2)]	−1.4138	−9.1893	<2.22e-16		
	θ_3_ [MA(3)]	−0.4371	−5.5881	7.6217e-12		

Table 2 showed that the models ARFIMA(0, 0.4941, 1), ARFIMA(0, 0.4941, 2), ARFIMA(0, 0.4941, 3), ARFIMA (0, 0.4941, 4), ARFIMA(1, 0.4941, 0), ARFIMA(1, 0.4941, 1), and ARFIMA(1, 0.4941, 3) were significant and suitable for building an ARFIMA model. However, not all significant models were applied in the subsequent steps. In order to identify the optimal model, a comparison was made between the AIC and BIC values. The evaluation of these values in Table 2 for the seven models revealed that the ARFIMA(1, 0.4941, 1) showed the lowest AIC and BIC values among the available alternatives. Consequently, it can be stated that the ARFIMA(1, 0.4941, 1) appeared as the most favorable choice.

Relying solely on model selection was insufficient to confirm that ARFIMA(1, 0.4941, 1) adequately fulfilled the necessary conditions as a time series. This led to the examination of the residual assumption of the ARFIMA(1, 0.4941, 1). Table 3 showed a test of residual assumptions for ARFIMA(1, 0.4941, 1).

**Table 3:** Residual assumption test of ARFIMA (1, 0.4941, 1) model.

**Residual assumption**		
	**Statistic** χ^2^	***p*** **-value**
Homoscedasticity	11.1380	0.0038
Autocorrelation	[0.1114, 3.8949]	>0.05
Normality	247.6700	<2.2e-16

Upon reviewing Table 3, it became evident that the *p*-value of the autocorrelation test had surpassed 0.05 at the 91st lag, indicating the absence of correlation among residuals. However, in the heteroscedasticity and normality tests, the *p*-values were below 0.05. This suggested the presence of heteroscedasticity or volatility effects on the residuals, necessitating their adjustment. The normality test could be overlooked due to the rapid fluctuations in the time series data. Therefore, ARFIMA(1, 0.4941, 1) was established as the best model with the following equation:


$$\begin{array}{l} {\left(1-B\right)}^{d}X_{t}=\Phi_{1}X_{t-1}+\theta_{1}\varepsilon_{t-1}+\varepsilon_{t}\\ {\left(1-B\right)}^{0.4941}X_{t}=0.6140X_{t-1}+0.3218\varepsilon_{t-1}+\varepsilon_{t} \end{array}$$

After obtaining ARFIMA, the presence of a heteroscedasticity effect in the residual ARFIMA prompted the need for an improved model to address this issue. Sections 3.2 and 3.3 focused on alternative models for addressing heteroscedasticity effects, including the linear model of Generalized Autoregressive Conditional Heteroscedasticity (GARCH) and nonlinear model of Long-Short Term Memory (LSTM).

### 3.2 Improving volatility residual ARFIMA using GARCH

**Figure 6:**
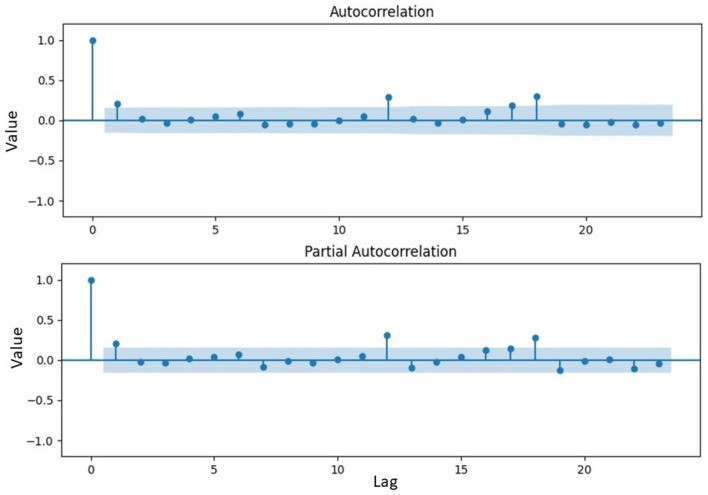
The ACF and PACF chart for determining the order of GARCH model.

According to the residual assumptions of ARFIMA(1, 0.4941, 1) shown in Table 3, the heteroscedasticity assumption had not been met. Consequently, an advanced model was necessary to enhance ARFIMA and minimize variance in the residuals. One traditional time series, GARCH, had been developed to counteract the random fluctuating variance or heteroscedasticity impact. The creation of a GARCH involved using ACF and PACF charts to determine the order of the model. Figure 6 showed the ACF and PACF charts for GARCH.

Figure 6 showed the significance of the ACF and PACF charts at lag 2. The initial conjecture for model orders *P* and *Q* had been combinations of 2 and 0, respectively. Eight potential models could be constructed using combinations including GARCH(0,1), GARCH(0,2), GARCH(1,0), GARCH(1,1), GARCH(1,2), GARCH(2,0), GARCH(2,1), and GARCH(2,2). The significant parameters had been identified based on the p-value, which had been lower than the significance level after estimating parameters for possible models using combinations from the ACF and PACF charts. Among these combinations, GARCH(0,1), GARCH(0,2), GARCH(1,0), GARCH(1,1), and GARCH(2,0) had been relevant parameters for constructing GARCH. Table 4 showed the estimated parameters for these models, along with their AIC and BIC values.

**Table 4:** The significant estimated parameters of the GARCH model with their AIC or BIC value.

**Model**	**Estimation parameter**				**The best model selection**		
	**Parameter**	**Estimate**	***z*** **-value**	**Pr (**>|*z*|**)**	**AIC**	**BIC**	**HQ**
GARCH(0,1)	β_1_	0.9880	809.1039	0.0000	−6.9013	−6.8631	−6.8858
GARCH(0,2)	β_1_	0.4433	10.2208	0.0000	−6.8736	−6.8165	−6.8504
	β_2_	0.5329	12.4415	0.0000			
GARCH(1,0)	α_1_	0.9989	20.9141	0.0000	−7.0468	−7.0087	−7.03137
GARCH(1,1)	α_1_	0.7335	4.0984	4.1600e-05	−7.0373	−6.9802	−7.0141
	β_1_	0.2655	6.4339	1.2400e-10			
GARCH(2,0)	α_1_	0.9905	5.2035	1.9600e-07	−7.0506	−6.9934	−7.0274
	α_2_	0.0085	1.9832	0.04735			

The optimal model was determined by selecting those with the lowest AIC or BIC value from among several potentially significant ARFIMA model. The smallest values for AIC, BIC, and HQ were identified from Table 4 as GARCH(1, 0), GARCH(2, 0), and GARCH(1, 0), respectively. Since GARCH(1, 0) had the lowest AIC and HQ values, it was selected to enhance the residual ARFIMA model and address the issue of volatility. The residual GARCH(1, 0) ARFIMA(1, 0.4941, 1) model could be expressed as follows:


(26) $$\begin{array}{l} \sigma_{t}^{2}=\alpha_{1}\varepsilon_{t-1}^{2}+\epsilon_{t}^{*}=0.9989\varepsilon_{t-1}^{2}+\epsilon_{t}^{*} \end{array}$$

where ε_*t*_ = σ_*t*_*e*_*t*_, *e*_*t*_ ~ *N*(0, 1), and $\epsilon_{t}^{*}$ as the residual of the GARCH model. By combining the ARFIMA and GARCH models, this new model of ARFIMA(1, 0.4941, 1)-GARCH(1,0) served as a potential alternative for forecasting future inflation rates.

### 3.3 Improving volatility residual ARFIMA using LSTM

After fitting ARFIMA to the long-patterned inflation data series, efforts were directed toward improving the heteroscedasticity of the model by addressing the residual. Visual diagnosis could be used to identify the presence of the heteroscedasticity effect. Outlier data indicated that an advanced model was necessary to adjust the effect due to data variability. Modifying the residual heteroscedasticity effect of ARFIMA was essential. The persistent vanishing/exploding gradient problem resulting from long-term dependencies, even with substantial data, posed a challenge due to the random fluctuation in residuals. Consequently, the application of LSTM neural network was deemed necessary (Shewalkar et al., 2019).

**Figure 7:**
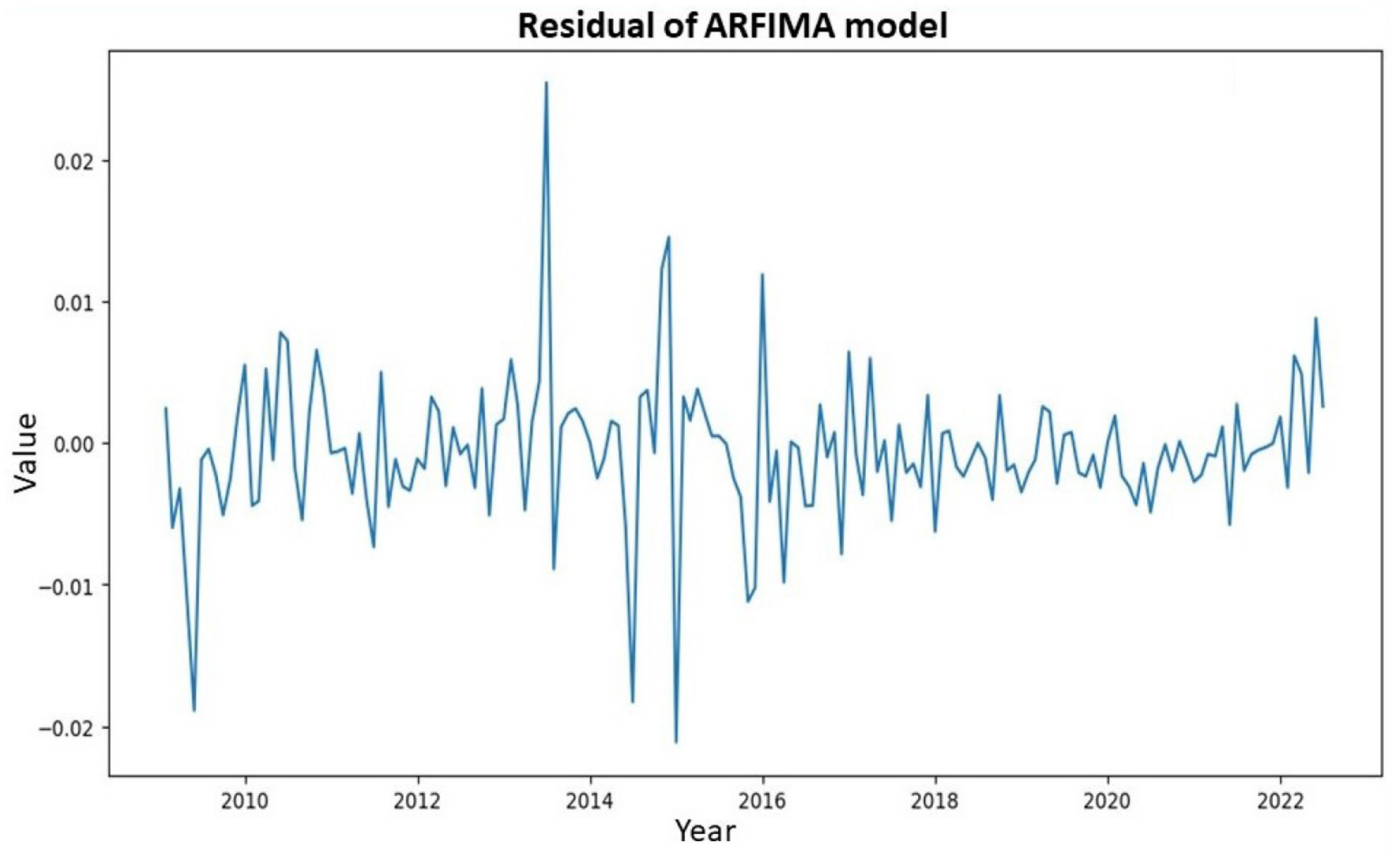
Historical residual of ARFIMA model.

The original residual of ARFIMA initially applied to inflation data from February 2009 to July 2022 was shown in Figure 7. This presentation aimed to provide insights into the fluctuation patterns of the residual model.

**Figure 8:**
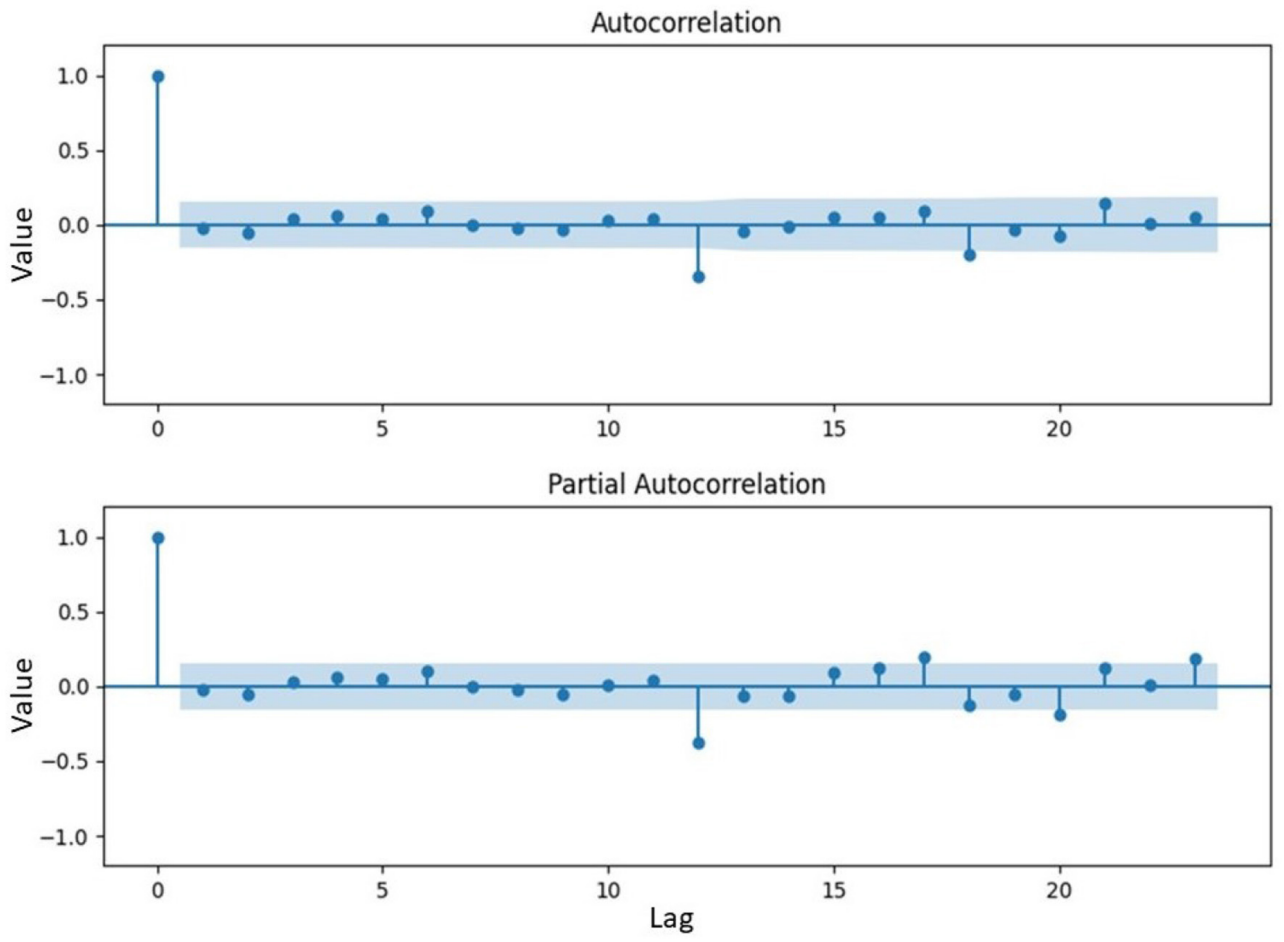
The ACF and PACF plot of residual data of ARFIMA model.

Analysis of Figure 7 showed that the residual ARFIMA model continued to exhibit considerable fluctuations, indicating the persistence of volatility in the residual data. However, the residual ARFIMA became stationary concerning the mean value, with the data fluctuating around zero. This was the reason a nonlinear model was considered for improving the residual data of ARFIMA. The performance level would then be compared with the linear GARCH discussed earlier. Figure 8 showed the ACF and PACF of the processed data.

The ACF and PACF plots in Figure 8 showed a significant impact at the twelfth lag. This observation implied that the inflation in each year was dependent on the value of the next year. Consequently, a preferred model aimed at mitigating the heteroscedasticity effect would involved employing 12 as the number of significant lags. To enhance the residual of ARFIMA, LSTM neural network, which was a nonlinear model, would be applied. This model included training the network on 80% of the dataset and testing it on the remaining 20% to evaluate performance. Parameter settings for LSTM model were clearly shown in Table 5.

**Table 5:** Parameter settings for LSTM model.

**Parameters**	**Values**
Total layers	4
The number of lags	12
The number of neurons	(16, 32, 64, 128)
Learning rate	0.001
Optimization approach	Adam
Size of batch	32
Total repetition (epochs)	300
Training data	118
Testing data	44

**Figure 9:**
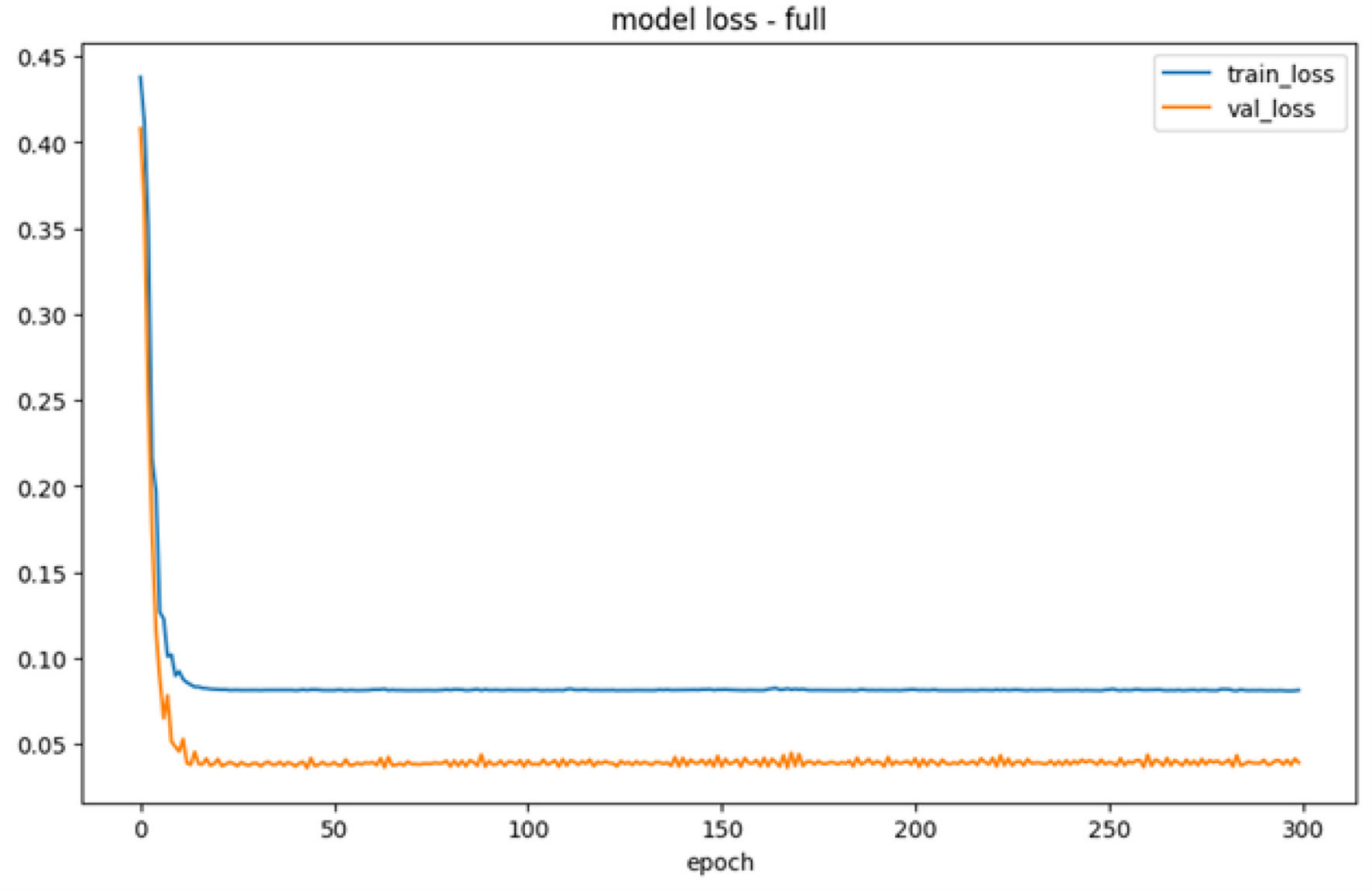
Validation error.

During the data processing, the model loss for each training and testing data was shown in Figure 9.

Figure 9 showed the training process, which exhibited a substantial decrease in error from 0.45 to ~0.10 on LSTM neural network. However, during testing, the output error proved to be lower than those observed during training. The testing process indicated a strong reduction in the output error from 0.42 to ~0.05, surpassing the performance of the training process. Consequently, validation suggested that the error in testing would be smaller compared to the training. Based on the result, the constructed LSTM neural network effectively recognized new data and reached the actual values through numerical processing.

In determining the sensitivity of random initial weights of LSTM model and parameters of GARCH model, five random experiments will be executed. The results of five experiments will be shown in following Table 6.

**Table 6:** Sensitivity evaluation inflation model of Indonesia.

**Experiments**	**ARFIMA-GARCH**			**ARFIMA-LSTM**		
	**Mean**	**Variance**	**Standard error**	**Mean**	**Variance**	**Standard error**
Actual data	0.0428	0.0004	0.0193	0.0428	0.0004	0.0193
1.	0.0406	0.0003	0.0171	0.0427	0.0004	0.0191
2.	0.0406	0.0003	0.0171	0.0458	0.0004	0.0190
3.	0.0406	0.0003	0.0171	0.0428	0.0004	0.0189
4.	0.0406	0.0003	0.0171	0.0457	0.0004	0.0190
5.	0.0406	0.0003	0.0171	0.0438	0.0004	0.0190

Table 6 shows sensitivity random initial weights against the actual data. Based on Table 6, all five experiments has the values that approach measures of dispersion actual values: mean, variance, or standard error. It concludes that LSTM has the best performance to capture the long-pattern data and fluctuation of the inflation data because ARFIMA-LSTM has better performance than ARFIMA-GARCH to approach the measures of dispersion inflation data. The results also show that the ARFIMA-GARCH doesn't change for each experiments since this hybrid model use Newton Raphson to approach the optimal parameters using high dimensional matrices.

### 3.4 Evaluating the volatility model

**Figure 10:**
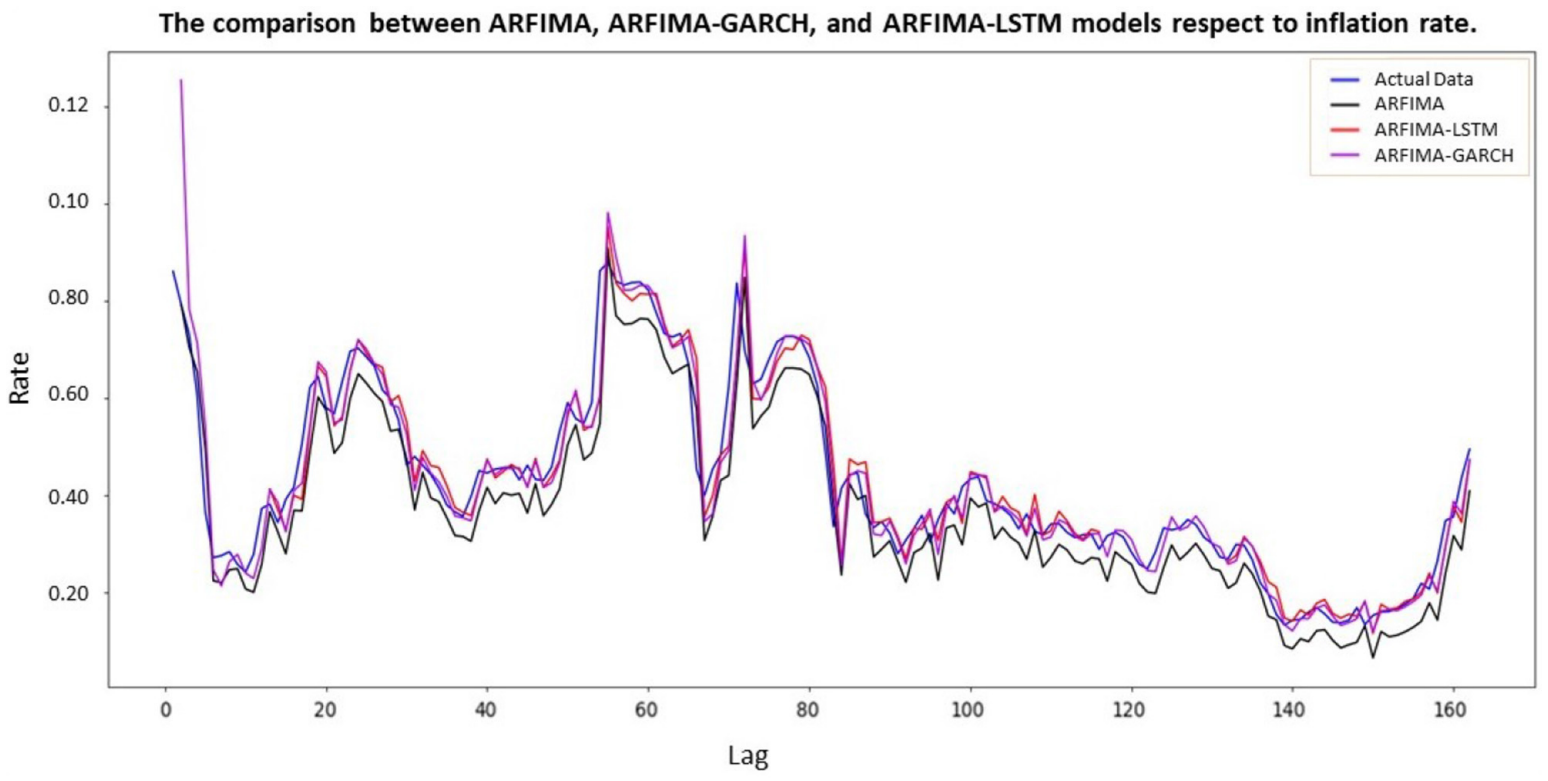
Comparison between actual data of inflation and ARFIMA-LSTM model.

After adjusting the heteroscedasticity effect within the residual ARFIMA, a comparison of ARFIMA-GARCH and ARFIMA-LSTM models was conducted to evaluate their performance. Figure 10 graphically showed this comparison, including the actual inflation rate data, ARFIMA(1, 0.4941, 1), ARFIMA(1, 0.4941, 1)-LSTM, and ARFIMA(1, 0.4941, 1)-GARCH(1, 0). These four representations were respectively depicted in blue, black, red, and purple. A model that closely associated with the actual value and accurately tracked its movements would likely be more accurate.

The black, red, and purple lines in Figure 10 represented ARFIMA, ARFIMA-LSTM, and ARFIMA-GARCH models, respectively. These lines smoothly estimated the blue line (actual value) using a numerical model to imitate and recognize the actual value. This indicated that the improved models represented by the red and purple lines closely approximated the blue line, surpassing the accuracy of the black line. Essentially, the red line performed better than the purple line in estimating the blue line. In other words, the sensitivity of residual data, representing volatility data, could be effectively handled through the application of numerical models, specifically LSTM neural network. The validity of this statement was supported by evaluating the model using metrics such as MSE, MAE, and MAPE. A comparison between ARFIMA and ARFIMA-LSTM could be seen in Table 7.

**Table 7:** Evaluation inflation model in Indonesia.

**Model**	**MAE**	**MSE**	**MAPE**
ARFIMA	0.0063	5.8901 e-05	16.4297
ARFIMA-GARCH	0.0043	4.0726 e-05	9.7766
ARFIMA-LSTM	0.0039	3.3326 e-05	9.5185

From Table 7, ARFIMA-LSTM model yielded the smallest values for all three evaluation criteria. This outcome suggested that employing the numerical model of LSTM neural network enhanced and refined the predicted inflation values. After adjusting the long memory pattern of inflation data, the residuals of ARFIMA went through further processing using LSTM to address the vanishing gradient issue inherent in ARFIMA volatility component, often referred to as heteroscedasticity effects. Consequently, the preferred LSTM neural network effectively improved the heteroscedasticity issue of the classical ARFIMA. Mitigating the impact of heteroscedasticity was achieved through either linear or nonlinear models (Devianto et al., 2023). The application of GARCH served as a linear model, while using the Feed Forward Neural Network (FFNN) represented the nonlinear. Among the models, FFNN appeared to be the most effective for addressing heteroscedasticity when compared to GARCH and hybrid GARCH-FFNN. The results in Table 7 also showed that the neural network employing LSTM outperformed the classical GARCH in terms of nonlinear modeling. The same scenario is also obtained in the financial market, where the rapid development of artificial intelligence (LSTM) allows for a more accurate prediction of financial market volatility than the baseline model of GARCH (Liu et al., 2022). The results also underscored the optimal performance achieved through the fusion of the neural network and ARFIMA, particularly when applied to inflation data. The assessment was based on metrics including MSE, MAE, and MAPE.

Based on Table 7 that tells about inflation data in Indonesia, the United States inflation model is also built employing ARFIMA-GARCH and ARFIMA-LSTM. Analogically, the inflation evaluation models in the United States are shown in Table 8. Table 8 represents the evaluation model of ARIMA, ARIMA-GARCH, and ARIMA-LSTM models of inflation data. Based on Table 8, the ARIMA-LSTM is still having the best performance in all proposed model of inflation data in United States. However, the ARIMA-GARCH does not give the better performance than the classical model of ARIMA in adjusting the error but it can do adjustment of the hetroscedasticity effect.

**Table 8:** Evaluation inflation model in United States.

**Model**	**MAE**	**MSE**	**MAPE**
ARIMA	0.0015	4.4778E-06	8.3694
ARIMA-GARCH	0.0023	12.9270E-06	13.0368
ARIMA-LSTM	0.0012	3.5897E-06	6.8719

Based on these two cases about the modeling of inflation data in Indonesia and United States, the hybridization between the classical model and LSTM method gives the best performance in adjusting the heteroscedasticity effects and also the error of the classical model of ARFIMA for inflation data in Indonesia and ARIMA for inflation data in United States. In addition, the fractional time series data modeling can be applied if the series data has the long-memory pattern data.

## 4 Conclusion

In conclusion, this paper proposed an enhanced sensitivity model by incorporating Long Short-Term Memory (LSTM) neural network and Generalized Autoregressive Conditional Heteroscedasticity (GARCH) into a long-memory model of ARFIMA. To achieve stability, the GARCH model will reconstruct the volatility in residual of ARFIMA using numerical processing of Newton Raphson and the LSTM will reconstruct the volatility in residual of ARFIMA using the adjustment of random initial weights until the threshold error is obtained. Recognizing the limitations of the classical long-memory ARFIMA in accurately predicting inflation, this study underscored the necessity for ARFIMA-LSTM and ARFIMA-GARCH models.

The proposed models, ARFIMA-LSTM and ARFIMA-GARCH, are then compared by using Mean Absolute Error (MAE), Mean Square Error (MSE), and Mean Absolute Percentage Error (MAPE). The results show that the ARFIMA-LSTM and ARFIMA-GARCH improve the error and also the heteroscedasticity effect of the classical ARFIMA model. In addition, the advantages of ARFIMA-LSTM are to achieve of the stability by learning the previous data with the dynamical system will increase the complexity in processing the networks, to approximate the gradient of ARFIMA through numerical computations until a defined threshold error was met, to retain information and patterns in residual data caused LSTM to effectively mitigate the heteroscedasticity issue present in ARFIMA. Despite capturing the long-pattern data inherent in inflation, the ARFIMA model does not adequately optimize inflation prediction. This led to the investigation of a nonlinear solution to the gradient problem. The method included using the LSTM, which is known for its ability to retain information and patterns in residual data. Due to this implementation, LSTM effectively mitigated the heteroscedasticity problem in ARFIMA. As a result, the model handled the vanishing gradient problem, allowing the LSTM neural network to learn and bridge considerable temporal gaps even spanning more than 1,000 discrete time steps.

However, the randomness of initially weights and the number of iterations persist the limitations of the ARFIMA-LSTM model, with the number of iterations increasing as the threshold error of the networks decreases. It will require an extended period to determine a suitable weighting parameter. Furthermore, because the initial weights are random, the ideal parameters do not have the same values across experiments, necessitating validation of the training data while developing networks. If the error of the training data is less than the error of the testing data, the networks are required to be stopped and the processing repeated until the error of the training data is more than the error of the testing data to ensure that the networks can recognize the new data using the obtained model of ARFIMA-LSTM.

## Data availability statement

The datasets presented in this study can be found in online repositories. The names of the repository/repositories and accession number(s) can be found at: https://www.bi.go.id/id/statistik/indikator/data-inflasi.aspx.

## Author contributions

EA: Formal analysis, Investigation, Validation, Writing—original draft. EH: Formal analysis, Data curation, Writing—review & editing. DD: Formal analysis, Conceptualization, Investigation, Methodology, Validation, Writing—original draft. MY: Formal analysis, Methodology, Software, Visualization, Writing—original draft. DP: Data curation, Formal analysis, Methodology, Writing—review & editing.
